# Economic analysis: PICC versus short catheter for prolonged home antibiotic therapy

**DOI:** 10.15649/cuidarte.4124

**Published:** 2025-05-01

**Authors:** Mariana Vélez-Bonilla, Catalina Hernández-Flórez, Allan Solano-Felizzola, Sandra B Amado-Garzón, Diego Rosselli

**Affiliations:** 1 Department of Internal Medicine – Hospital Universitario San Ignacio, Bogotá, Colombia. E-mail: velez.mariana@javeriana.edu.co Hospital Universitario San Ignacio Bogotá Colombia velez.mariana@javeriana.edu.co; 2 Department of Internal Medicine – Hospital Universitario San Ignacio, Bogotá, Colombia. Hospital at home. Medicine – Hospital Universitario San Ignacio, Bogotá, Colombia E-mail: chernandezf@husi.org.co Hospital Universitario San Ignacio Bogotá Colombia chernandezf@husi.org.co; 3 Hospital at home. Medicine – Hospital Universitario San Ignacio, Bogotá, Colombia. E-mail: asolano@husi.org.co Hospital Universitario San Ignacio Bogotá Colombia asolano@husi.org.co; 4 Department of Internal Medicine – Hospital Universitario San Ignacio, Bogotá, Colombia. E-mail: sbamado@husi.org.co Hospital Universitario San Ignacio Bogotá Colombia sbamado@husi.org.co; 5 Department of Clinical Epidemiology and Biostatistics – Pontificia Universidad Javeriana, Bogotá, Colombia. E-mail: drosselli@javeriana.edu.co Pontificia Universidad Javeriana Bogotá Colombia drosselli@javeriana.edu.co

**Keywords:** Outpatients, Catheterization Peripheral, Catheters, Catheter-Related Infections, Deep Vein Thrombosis, Pacientes Ambulatorios, Cateterismo Periférico, Catéteres, Infecciones Relacionadas con Catéteres, Trombosis de Vena Profunda, Pacientes Ambulatoriais, Cateterismo Periférico, Catéteres, Infecções Relacionadas a Cateter, Trombose de Veia Profunda

## Abstract

**Introduction:**

Hospital-at-home programs rely on vascular access devices for secure administration of parenteral antimicrobials. While guidelines recommend peripherally inserted central catheters (PICC) for treatments ≥14 days, short peripheral catheters (SPC) are often used instead. Cost-effectiveness studies comparing these devices and their complications are limited.

**Objective:**

This study conducted an economic evaluation comparing PICC and SPC for patient outpatient parenteral antibiotic therapy.

**Materials and Methods:**

A literature review of catheter complication frequencies yielded 1053 papers, narrowed to 18 after independent peer review. Experts were consulted, and a list of items required for catheter use was compiled to determine costs. A decision tree model was developed based on complication frequencies and costs. Results were analyzed using incremental cost-effectiveness ratios (ICER), univariate sensitivity analysis (tornado diagram), and multivariate sensitivity analysis (Monte Carlo simulation).

**Results:**

Major complications were similar between devices, but minor complications were more frequent with SPC. The PICC reference case assumed 50%-50% radiologist/nurse insertion, catheter cost ($74,7), ≤15-day treatment, and complication prevalence. Higher costs associated with PICC were linked to catheter material and radiologist insertion. Multivariate analysis showed ICERs of $49,2 with 90% nurse-led insertion and $24,3 with 100% nurse-led insertions, assuming a 50% PICC price reduction.

**Discussion:**

PICC was more effective in reducing minor complications. Costs decreased with nurse-led insertions and lower catheter material costs.

**Conclusion:**

Increasing PICC use for extended treatments could reduce overall costs and lower ICERs, highlighting their potential economic advantage despite higher initial expenses.

## Resumen

**Introducción:**

Los programas de hospitalización domiciliaria dependen de dispositivos de acceso vascular para administrar antimicrobianos parenterales. Aunque las guías recomiendan catéteres centrales de inserción periférica (PICC) para tratamientos de ≥14 días, se utilizan con frecuencia catéteres periféricos cortos (SPC). Los estudios de costo-efectividad que comparen estos dispositivos y sus complicaciones son limitados.

**Objetivo:**

Evaluar económicamente el uso del PICC y el SPC en pacientes con terapia antimicrobiana parenteral ambulatoria.

**Materiales y métodos:**

Se revisaron 1053 artículos sobre complicaciones relacionadas con catéteres, seleccionando 18 tras revisión por pares. Se consultó a expertos y se elaboró una lista de insumos para calcular costos. Se desarrolló un modelo de árbol de decisión con frecuencias de complicaciones y costos, utilizando análisis de costo-efectividad incremental (ICER), sensibilidad univariada (diagrama de tornado) y multivariada (simulación de Monte Carlo).

**Resultados:**

Las complicaciones mayores fueron similares entre dispositivos, pero las menores fueron más frecuentes con el SPC. El PICC presentó costos más altos asociados al material y a inserciones realizadas por radiólogos. El análisis multivariado mostró ICER de $49,2 con un 90% de inserciones por enfermeras y de $24,3 con un 100%, asumiendo una reducción del 50% en el precio del PICC.

**Discusión:**

Los PICC parecen más efectivos en la prevención de complicaciones menores. Las inserciones por enfermería y la reducción del costo del material del catéter podrían disminuir significativamente los costos totales.

**Conclusiones:**

Incrementar el uso del PICC para tratamientos prolongados podría reducir costos y mejorar la costo-efectividad, siendo más económico pese a costos iniciales más altos.

## Resumo

**Introdução:**

Programas de hospital em casa dependem de dispositivos de acesso vascular para administração segura de antimicrobianos parenterais. Enquanto as diretrizes recomendam Peripherally Inserted Central Catheters (PICC) para tratamentos ≥14 dias, Short Peripheral Catheters (SPC) são frequentemente usados. Estudos de custo-efetividade comparando esses dispositivos e suas complicações são limitados.

**Objetivo:**

Este estudo conduziu uma avaliação econômica comparando PICC e SPC para terapia antibiótica parenteral ambulatorial de pacientes.

**Materiais e métodos:**

Uma revisão de literatura das frequências de complicações do cateter rendeu 1.053 artigos, reduzidos para 18 após revisão por pares independente. Especialistas foram consultados e uma lista de itens necessários para o uso do cateter foi compilada para determinar os custos. Um modelo de árvore de decisão foi desenvolvido com base nas frequências e custos das complicações. Os resultados foram analisados usando Incremental Cost-Effectiveness Ratios (ICER), análise de sensibilidade univariada (diagrama de tornado) e análise de sensibilidade multivariada (simulação de Monte Carlo).

**Resultados:**

As complicações maiores foram semelhantes entre os dispositivos, mas as complicações menores foram mais frequentes com o SPC. O caso de referência do PICC assumiu 50%-50% de inserção por radiologista/enfermeiro, custo do cateter ($74,7), tratamento ≤15 dias e prevalência de complicações. Custos mais altos associados ao PICC foram vinculados ao material do cateter e à inserção do radiologista. A análise multivariada mostrou ICERs de $49,2 com 90% de inserção liderada por enfermeiros e $24,3 com 100% de inserção liderada por enfermeiros, assumindo uma redução de 50% no preço do PICC.

**Discussão:**

O PICC foi mais eficaz na redução de complicações menores. Os custos diminuíram com inserções lideradas por enfermeiros e menores custos com material do cateter.

**Conclusões:**

Aumentar o uso do PICC para tratamentos prolongados pode reduzir os custos gerais e diminuir os ICERs, destacando seu potencial vantagem econômica, apesar das maiores despesas iniciais.

## Introduction

Hospital-at-home (HaH) programs enable the treatment of infectious diseases through outpatient parenteral antimicrobial therapy (OPAT), which largely corresponds to prolonged courses (>14 days)^1^,^2^. This is why an adequate vascular access device (VAD) is necessary to minimize the rate of catheter-associated complications^2^-^4^. 

International guidelines for selecting the most appropriate VAD consider various factors, such as compatibility with peripheral veins, staff expertise in insertion and maintenance, the condition of the patient's superficial venous network, the presence of chronic kidney disease (stage 3b, 4, or 5), and the duration of treatment^2^,^5^-^7^. For prolonged OPAT or when solutions are not suitable for peripheral administration, the guidelines recommend using a peripheral inserted central catheter (PICC). PICCs provide central venous access, can be inserted by nurses, offer patient satisfaction, and allow for easy care, making them suitable for home-based use^5^,^7^,^8^. 

It is common to use the short peripheral catheter (SPC) in hospitalized patients, with some registries indicating it as the most utilized VAD^9^. Although this catheter is less expensive and easier to insert, complication rates of up to 59% have been reported, often leading to catheter failure^10^. These complications can be major, such as deep vein thrombosis (DVT) and catheter-associated bloodstream infection (CRBSI), as well as minor, including phlebitis, local infections, occlusion, extravasation, infiltration, and displacement or accidental removal^4^,^10^. The necessity for a new catheter insertion increases the risk of further failures, impacts the patient's venous network, causes pain, reduces patient satisfaction levels, prolongs care, and results in additional costs^10^. 

Available literature reports a lower rate of minor complications and reduced use of catheters and venipunctures during treatment with PICC compared to SPC, resulting in a lower rate of catheter failure with PICCs^11^,^12^; however, this benefit is offset by the higher cost of consumables associated with PICC use^13^-^16^. As for major complications, there is a higher tendency to report them with PICCs in the in-hospital setting. Nevertheless, in the outpatient setting, there appears to be a lower frequency of CRBSI (0.04 per 1000 catheter-days), as well as reduced rates of infections and thrombosis^8^,^13^,^17^,^18^. 

In Colombia, some studies have assessed the complication rates associated with VADs^19^, including evidence confirming a lower frequency of CRBSI in HaH settings when using PICCs^20^. However, no studies evaluate the cost-effectiveness of using PICCs versus SPCs for prolonged OPAT regarding the complications associated with each device. This study aims to support more informed decision-making on allocating resources for VAD use. 

## Materials and Methods

An economic evaluation study was conducted to assess the relationship between safety (i.e., the occurrence of complications) and the costs associated with two VAD options for prolonged OPAT in adults receiving HaH care. The methodology followed the Colombian Health Technology Manual (IETS, for the Spanish acronym) guidelines for economic evaluation studies^21^ and adhered to the recommendations of the Consolidated Health Economic Evaluation Reporting Standards (CHEERS) 2022^22^. 

The time horizon for the analysis corresponded to the duration of antimicrobial therapy (a minimum of 14 days), considering that VAD-related complications occur during the catheter's dwell time. The third-party payer's cost perspective was considered, evaluating direct medical costs associated with the use of the devices, required supplies, as well as the cost of any complications that occur. A discount rate was not applied, as the time horizon was less than 12 months. Approval from the institutional ethics board was obtained, and no patient data were used. No funding was received for this study. 

**Outcomes**

Complications were classified as minor (phlebitis, occlusion, extravasation, dislodgement, and superficial vein thrombosis [SVT]) and major (DVT, CRBSI)^13^,^23^. To determine their frequencies, a comprehensive literature search was performed in the PubMed, Embase, CINAHL, LILACS, and Scopus databases without year restrictions. The search was limited to studies published in English or Spanish and included comparative, analytical, observational, and cross-sectional studies involving adult populations in home and/or in-patient settings, with exposure to PICC and/or SPC as the VAD. Studies involving pediatric populations, intensive care unit hospitalization, case reports, case series, and the use of VADs for chemotherapy administration were excluded. For study selection, an independent screening of the documents according to title and abstract was performed by two authors, with a third reviewer resolving any disagreement. The Rayyan - Intelligent Systematic Review tool was used^24^, and a full- text review of the articles was subsequently conducted. Data extraction was then performed using a database designed in Microsoft Excel to capture the highest quality of evidence for each complication. 

Subsequently, focus group meetings with experts from disciplines involved in the management of device-related complications were held. The aim was to contrast the findings from the literature with the reality and experience of each group and to compile the inputs, diagnostic methods, and therapeutic methods necessary for managing each complication. The distribution was as follows: for minor complications, a focus group was conducted with three experts in vascular access nursing and interventional radiology; for thrombotic complications, information was gathered from the institution's anticoagulation clinic group; and for infectious complications, meetings were held with experts in infectious diseases. 

**Cost evaluation**

Based on the information gathered from the focus groups, all diagnostic and therapeutic tools for using each VAD (insertion, maintenance, and complications) were grouped. A unit cost was assigned to each of these inputs using information from the institution’s purchasing department and the institution's charge rate conditions, obtaining the charge rates from an insurer. The costs of inserting a new device were not included, as the device is not always removed in the event of complications, and even when it is removed, a new device is not always inserted. All costs were estimated in 2022 Colombian pesos (COP) and converted to U.S. dollars using the average exchange rate for that year (1 USD= 4,255 COP). 

**Modeling and analysis**

A decision tree model was designed using the TreeAge Pro2015 software tool (TreeAge Software, Inc., Williamstown, MA). For each VAD, branches were constructed, including probability nodes with the frequencies of major and minor complications, along with the associated costs. The results were measured using the incremental cost-effectiveness ratio (ICER) (PICC cost - SPC cost / complication avoided with PICC - SPC). A univariate sensitivity analysis was performed using a tornado diagram, and a multivariate sensitivity analysis was conducted using Monte Carlo simulation. The dataset is available in Mendeley Data^25^. 

## Results

**Literature search for complications**

The initial search identified 1053 references. Three were obtained from additional sources, including expert recommendations, reference lists, and gray literature. The final selection process included 18 articles. Of these, 2 evaluated PICC-related CRBSI^17^,^26^, 3 evaluated PICC-associated DVT^27^-^29^, 2 explored local infection and phlebitis^30^,^31^, and 5 various outcomes related to PICC use^8^,^11^-^13^,^32^. Regarding SPCs, 1 article evaluated the presence of CRBSI33, and 6 evaluated the other outcomes^10^,^13^,^34^-^37^. For the selection of the source of the reference case for each outcome, the article with the best available quality of evidence and a reported range of frequencies was chosen and evaluated. Overall, the frequency of major complications for each catheter is similar, while minor complications were more frequent with SPCs than PICCs ( Table 1).

**Table 1:** Frequency of complications

VAD Complication	PICC			SPC		
	RC (%)	Range (%)	References	RC (%)	Range (%)	References
Major						
CRBSI	1.80	0-1.80	30,32	0.05	0.05-2.20	10, 33
DVT	1.50	0-3	27,30,32	3.40	3.40	13
Total	3.30	-	22,26,29	3.45	-	10,11, 31
Minor						
Local infection	2.90	2.90-5.70	12, 30	2.30	2.30	35
SVT	2.90	2.90-29	13, 28	44.80	44.80	13
Phlebitis	0.50	0.50-0.60	12, 30	19.30	7.70-32.20	33-35
Occlusion	3.20	0.90-5.80	8, 30,32	8	8-16.20	34,35
Dislodgement	6.50	6.10-6.50	8,12	6	6-8.40	34,35
Infiltration/extravasation	0	0	11	13.70	13.70-23.90	10,34
Total	16	-	8,11,23,26-30	94.10	-	10,11, 31-33

VAD: Vascular Access Device; RC: Reference case; PICC: Peripheral inserted central catheter; SPC: short peripheral catheter; CRBSI: Catheter-related bloodstream infection; DVT: Deep vein thrombosis; SVT: superficial vein thrombosis.

**Focus groups**

The list of inputs obtained through the focus groups is summarized in Supplementary Table 1. Regarding minor complications, the nursing focus group agreed with the frequencies found in the literature search. Considering thrombotic complications (DVT and SVT), the anticoagulation clinic group agreed with the frequencies reported by the reference case, and, finally, the infectious disease group supported the results found for CRBSI. 

**Costs, modeling, and analysis**

The total cost of using each VAD was calculated in COP according to the person responsible for insertion (nurse or interventional radiologist), maintenance, and the occurrence of each complication. The highest costs were associated with PICC insertion by interventional radiology ($281) and the cost of major complications with the use of both VADs (DVT: $207, CRBSI: $123.3) (Table S2). 

**Figure 1:**
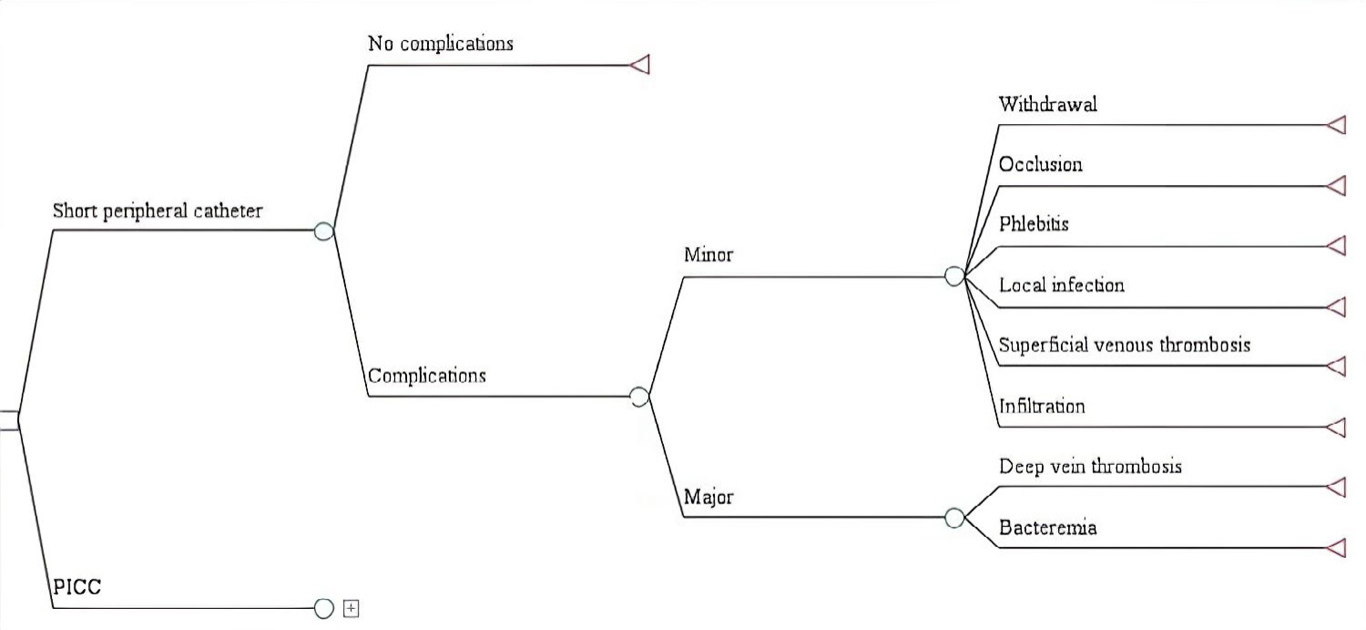
Decision tree model

**Figure 2:**
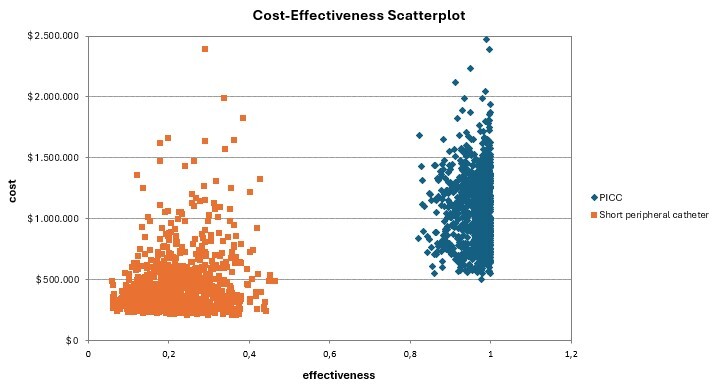
Cost-effectiveness Scatterplot

The reference case for PICC assumed 50.00% insertion by interventional radiology and 50.00% by nursing staff, a catheter price of $74.7, a minimum treatment duration of 15 days, and the complication frequencies outlined in Table 1. For SPC, the reference case included nursing-led insertion, a catheter price listed in Table S1, the same treatment duration as PICC, and the complication percentages reported in Table 1. In the decision tree model (Figure 1), the frequency of major complications was similar for both VADs (3.30% for PICC, 3.45% for SPC), which limited the analysis to minor complications, whose costs varied depending on the difference in the occurrence of each outcome. SVT and phlebitis associated with SPC use were the costliest minor complications (Table S2). In the reference case, the ICER per minor complication avoided with the use of PICC was $199. The cost-effectiveness scatterplot illustrating various catheter price scenarios is shown in Figure 2. 

*PICC: Peripheral inserted central catheter*

*PICC: Peripheral inserted central catheter*

**Sensitivity analysis**

**Figure 3:**
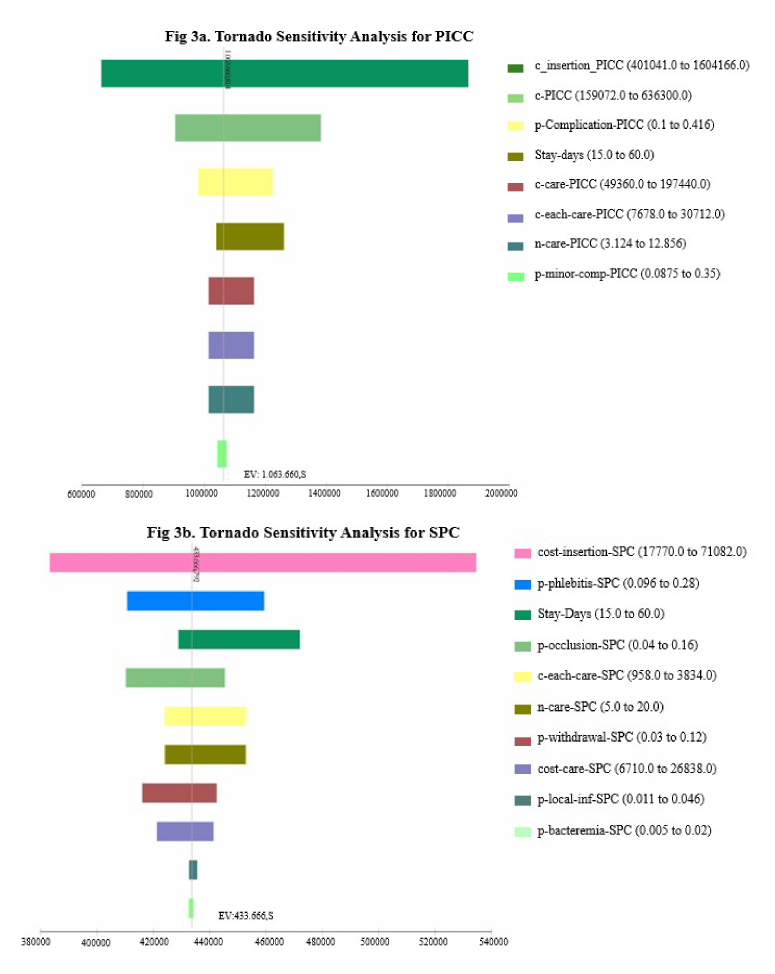
Univariate sensitivity analysis. Tornado graph for PICC (3a) and SPC (3b)

In the univariate sensitivity analysis of minor complications for PICC, the variables with the most significant influence on the overall cost were those related to catheter insertion costs. For SPC, the key factors affecting costs included insertion expenses, phlebitis-related costs, and the duration of hospital stay. These findings are illustrated in the tornado diagram (Figure 3a-b). 

Considering the catheter cost and the individual responsible for insertion, in the two-way sensitivity analysis, the most unfavorable scenario is PICC insertion in 100,00% of cases by interventional radiologists at the current catheter price, resulting in an ICER of $323.40. Conversely, with 100,00% nurse-led PICC insertion at the standard catheter price, the ICER to avoid a minor complication is $74.60. In more realistic scenarios, with 90,00% of nurse-led PICC insertions and a 30,00% reduction in PICC price, the ICER would be $69,40; it would be $49,2 with a 50,00% price reduction and $24,3 if 100,00% of catheters were inserted by nurses at that last price. 

## Discussion

In the literature review, the frequency of major complications is comparable between both VADs when the patient is at home, as the reported incidence of CRBSI and DVT is lower than in hospitalized patients, in some cohorts being close to 0%^8^,^11^,^17^,^30^ and reaching a frequency similar to that reported with SPC use^33^. 

The economic evaluation conducted in this study suggests that PICC is more effective than SPC in reducing the occurrence of minor complications, except for accidental withdrawal, which showed similar rates for both VADs. SVT and phlebitis were identified as the most common and costly complications associated with SPC use. Furthermore, an incremental cost of $199 was documented to avoid a minor complication with PICC. The univariate sensitivity analysis showed the influence of higher costs of PICC insertion and materials, aligning with the findings of Dychter et al.; however, complications were not considered in this review^38^. Likewise, a study by Periard et al.^13^ assessing the cost-effectiveness of both VADs showed higher costs associated with PICC due to the insertion materials. However, unlike our study, they evaluated efficacy based on the number of catheters and venipunctures needed for intravenous treatment. In contrast, a review of cost-effectiveness studies of PICC and SPC in the pediatric population found favorable outcomes for PICC use in short-term parenteral nutrition^39^. This highlights that, to the best of our knowledge, no economic evaluation studies have considered hidden post-insertion costs of VADs, such as maintenance and complications, in the adult population. 

Catheter failure rates are reported at 54-59% for SPC^10^,^40^ and 20% for PICC in two cohorts of patients undergoing long-term antibiotic treatment^11^,^31^. It is widely recognized that the risk of subsequent failures increases with each catheter failure due to factors like phlebitis, infiltration, extravasation, vascular quality, and history of previous venous punctures^40^. While our study did not account for this factor, given the complexity of cost and measurement implications, the univariate sensitivity analysis revealed that factors with the greatest economic impact for SPC included insertion cost, phlebitis, and length of hospital stay. These factors may align with a higher risk of subsequent failures and complications, given the longer exposure time. Considering the failure rate of PICCs, their use may be advantageous when evaluating total costs, especially in prolonged treatments, supporting international guidelines recommending PICC use for treatments exceeding 14 days^2^-^5^. 

Other potential hidden costs associated with extended treatments, such as transportation expenses for home care personnel, patient satisfaction, and venous depletion due to catheter failure, were not factored in this study due to challenges in cost assessment^10^. A randomized study comparing SPC and PICC use in treatments lasting more than 5 days showed higher levels of patient satisfaction with PICCs (96.8% vs. 79.3%)^13^. Despite the limitations in assessing patient satisfaction costs, whether this factor could indicate an overall benefit in prolonged parenteral treatment scenarios remains uncertain. 

Complementarily, we emphasize the two-way sensitivity analysis, which helped to determine the impact of VAD price variation and the person responsible for insertion on the modification of the ICER. With a higher proportion of insertions performed by nurses, the ICER tended to decrease, as did the cost of the catheter. In a realistic scenario where 100,00% of PICCs are inserted by nurses and the catheter price decreases by 50,00%, the ICER would drop to $24,3. This possibility is supported by a study showing a 93% success rate for PICCs inserted by vascular access nurses, with a 42% increase in costs (p<0. 01) when PICCs are inserted by radiologists, and higher patient satisfaction in the nursing group^41^. Another study in pediatric patients also demonstrated lower prices of nurse-led PICC insertion^42^. This highlights the role of nurse-led vascular access teams (VATs), recommended by the Infusion Nurses Society^6^ as a strategy to improve healthcare quality, seize the window of opportunity, reduce venous depletion, and potentially lower total cost, as shown by Cortés et al.^43^. These findings suggest that the increased institutional demand for PICC use, in line with VAD guidelines, could facilitate price negotiations, potentially leading to a reduction in ICER. 

Several opportunities for future research have been identified, particularly the evaluation of the costs associated with peripheral catheter failure and the potential for missed antibiotic doses in Hospital-at-Home programs. This would complement the hidden costs not accounted for in this study and highlight the importance of selecting the most appropriate vascular access device despite its initial cost. Another area worth investigating, though challenging to quantify in terms of cost, is the comparison of patient and caregiver satisfaction in the context of hospital-based home care services, specifically when comparing SPCs and PICCs. 

It is hoped that these findings will support similar institutions in selecting the most appropriate VAD for prolonged OPAT to minimize minor complications, catheter failure, and venous depletion. Similarly, promoting the development of vascular access teams that offer cost advantages during VAD insertion and ultimately lead to reduced overall patient care expenses is recommended. Nevertheless, each organization within the system should assess the value they place on preventing minor complications and subsequent catheter failure. 

## Conclusions

When comparing PICCs and SPCs for prolonged OPAT in HaH settings, this study suggests that PICCs are more effective in preventing minor complications and subsequent catheter failure. However, they remain more costly due to insertion and material expenses. 

Nurse-led PICC insertion and implementation of VATs within healthcare institutions could lower overall costs. 

With the growing need for PICCs in prolonged therapies, a reduction in incremental costs could be attained, promoting the adoption of this device for the benefit of patients and OPAT services. 

## Supplemental Material

**Table S1:** PICC and SPC insertion and maintenance inputs

Input	Cost (USD)
PICC	
Disposable cap	0.05
Disposable adult mouthpiece with elastic band	0.03
Sterile procedure gown	1.25
Sterile gloves	0.3
Sterile field 1.30 x 1.30	1.53
Chlorhexidine soap	0.58
Chlorhexidine alcohol	0.56
Pre-cut gauze	0.04
Syringe 10cc -21G x 1 1/2	0.06
Hypodermic needle 30 G x 1/2	0.02
Sterile compress 18 x18 in unit package	0.28
Peripherally inserted central catheter PICC 5Fr x50cm	74.77
Tegaderm™ IV advance dressing 8.5x11.5	1.1
Evo IQ infusion system	4.37
Buretrol® Ref Arc 7503	0.58
Microclave connector	0.5
StatLock™ catheter stabilization device	2.3
Sensi care skin barrier towels	0.57
Fixomull® fixation tape	4.68
Alcohol towel	0.02
SPC	
Tegaderm™ IV advance dressing 6,5x7cm box x100	0.45
Safety intravenous catheter 22G x1.16	0.6
Alcohol towel	0.02
Evo IQ infusion system	4.37
Buretrol® Ref. Arc 7503	0.58
Syringe 10cc -21G x 1 1/2	0.06
Microclave connector	0.5

USD: U.S. dollars. PICC: peripheral inserted central catheter. SPC: short peripheral catheter. IV: intravenous.

**Table S2:** Costs of PICC and SPC use

Category	Cost (USD)	
	PICC	SPC
Insertion	
Nursing	95.95	7.17
Interventional radiology	281	-
Maintenance ^a^		
Frequent care (48h)	25	17.7
Standard care	6.1	10.9
Complications		
Bacteremia ^b^	123.3	123.3
DVT	207	207
SVTc	59	59
Phlebitis	0.16	7.5
Local infection ^b^	90.1	90.1
Occlusion	0.64	0.33
Displacement ^b^	1.4	7.1
Extravasation	0	1.4^c^

USD: U.S. dollars. PICC: peripheral inserted central catheter. SPC: short peripheral catheter. DVT: deep venous thrombosis. SVT: superficial venous thrombosis. a. 30 days; b. Cost of a new device must be added; c. Cost of Doppler ultrasound.
